# Combining metabolic phenotype determination with metabolomics and transcriptional analyses to reveal pathways regulated by hydroxycarboxylic acid receptor 2

**DOI:** 10.1007/s12672-022-00503-3

**Published:** 2022-06-13

**Authors:** Philipp Rabe, Mareike Gehmlich, Anna Peters, Petra Krumbholz, Anders Nordström, Claudia Stäubert

**Affiliations:** 1 Rudolf Schönheimer Institute of Biochemistry, Faculty of Medicine, Leipzig University, Johannisallee 30, 04103 Leipzig, Germany; 2 Swedish Metabolomics Centre, Department of Forest Genetics and Plant Physiology, Swedish University of Agricultural Sciences, Linnaeus väg 6, 901 87 Umeå, Sweden

**Keywords:** Metabolite-sensing GPCR, Cancer metabolism, Metabolite profile, LC-MS, HCA_2_, GPR109A

## Abstract

**Background:**

The adaptation of cellular metabolism is considered a hallmark of cancer. Oncogenic signaling pathways support tumorigenesis and cancer progression through the induction of certain metabolic phenotypes associated with altered regulation of key metabolic enzymes. Hydroxycarboxylic acid receptor 2 (HCA_2_) is a G protein-coupled receptor previously shown to act as a tumor suppressor. Here, we aimed to unveil the connection between cellular metabolism and HCA_2_ in BT-474 cells. Moreover, we intend to clarify how well this metabolic phenotype is reflected in transcriptional changes and metabolite levels as determined by global metabolomics analyses.

**Methods:**

We performed both, siRNA mediated knockdown of HCA_2_ and stimulation with the HCA_2_-specific agonist monomethyl fumarate. Seahorse technology was used to determine the role of HCA_2_ in BT-474 breast cancer cell metabolism and its potential to induce a switch in the metabolic phenotype in the presence of different energy substrates. Changes in the mRNA expression of metabolic enzymes were detected with real-time quantitative PCR (RT-qPCR). Untargeted liquid chromatography-mass spectrometry (LC-MS) metabolic profiling was used to determine changes in metabolite levels.

**Results:**

Knockdown or stimulation of HCA_2_ induced changes in the metabolic phenotype of BT474 cells dependent on the availability of energy substrates. The presence of HCA_2_ was associated with increased glycolytic flux with no fatty acids available. This was reflected in the increased mRNA expression of the glycolytic enzymes PFKFB4 and PKM2, which are known to promote the Warburg effect and have been described as prognostic markers in different types of cancer. With exogenous palmitate present, HCA_2_ caused elevated fatty acid oxidation and likely lipolysis. The increase in lipolysis was also detectable at the transcriptional level of ATGL and the metabolite levels of palmitic and stearic acid.

**Conclusions:**

We combined metabolic phenotype determination with metabolomics and transcriptional analyses and identified HCA_2_ as a regulator of glycolytic flux and fatty acid metabolism in BT-474 breast cancer cells. Thus, HCA_2_, for which agonists are already widely used to treat diseases such as psoriasis or hyperlipidemia, may prove useful as a target in combination cancer therapy.

**Supplementary Information:**

The online version contains supplementary material available at 10.1007/s12672-022-00503-3.

## Background

Cellular metabolism is tightly controlled to balance the demands for energy generation and biosynthesis. The main fuels for cellular energy generation are glucose (Glc), fatty acids (FAs) and amino acids such as glutamine (Gln). The regulation of enzymatic activity by phosphorylation, allosteric effectors or protein degradation is well described for central metabolic pathways such as glycolysis, the tricarboxylic acid (TCA) cycle, fatty acid oxidation (FAO) and fatty acid synthesis (FAS) [1, 2]. These processes are often controlled by extracellular signals activating specific receptors. G protein-coupled receptors (GPCRs) constitute the largest and most versatile family of membrane-spanning proteins [3]. They are involved in every physiological process, and 35% of all currently approved drugs act at GPCRs [4]. Hydroxycarboxylic acid receptor 2 (HCA_2_) is a Gα_i_-coupled receptor activated by the ketone body 3-hydroxybutyrate, which is produced by liver mitochondria and elevated in vivo under prolonged fasting conditions [5, 6]. HCA_2_ is expressed in adipocytes, where its activation causes an inhibition of lipolysis [7], and in immune cells in which the receptor is associated with anti-inflammatory responses [8]. Several lipid-lowering drugs, such as nicotinic acid, act as agonists of HCA_2_ [9]. Stimulation of HCA_2_ by nicotinic acid was shown to induce the abovementioned anti-inflammatory effects in a variety of cell types, such as macrophages and neutrophils (reviewed in [9]). Moreover, monomethyl fumarate (MMF), the active metabolite of the psoriasis and multiple sclerosis (MS) drug dimethyl fumarate, mediates some of its effects, such as reduced neutrophil adhesion, migration and recruitment, through HCA_2_ activation (reviewed in [10]). In addition to its physiological role, several studies suggest that HCA_2_ acts as a tumor suppressor in different types of cancer [11–13]. The expression of HCA_2_ in human breast cancer cells induced apoptosis and inhibited mammary tumor growth [11]. Furthermore, HCA_2_ is silenced in human colon cancer and colon cancer cell lines due to its tumor suppressive function [13]. Mutations in oncogenes and tumor suppressor genes regulate the expression and activity of metabolic enzymes [14], which subsequently results in metabolic alterations that may become visible/exploitable through metabolic profiling and biomarker discovery via liquid chromatography-mass spectrometry (LC-MS). Metabolomics aim to identify biomarkers that may prove useful for the diagnosis, monitoring and therapy of cancer [15].

The goal of the present study was to determine the role of HCA_2_ as an exemplary GPCR in the regulation of the metabolic phenotype in the presence of different energy substrates. In all the experiments, we used the BT-474 breast cancer cell line, for which we have previously shown the endogenous expression of HCA_2_ [16]. We performed metabolic analyses using Seahorse technology but additionally applied metabolic profiling using LC-MS and analyzed the transcriptional changes of selected central metabolic enzymes. Our study aimed to clarify whether a combination of these methods enables the determination of a distinct metabolic phenotype that is associated with the role of HCA_2_ in BT-474 cells as a representative cell line. This approach may be used to interpolate a combination of potential biomarkers associated with the functions of other genes.

## Methods

### Cell culture

The human embryonic kidney cell line HEK-293T (ATCC CRL-3216) and the human breast cancer cell line BT-474 (ATCC HTB-20) were obtained from the American Type Culture Collection. All cells were maintained at 37 °C in a humidified 5% CO_2_ incubator, and 10% fetal bovine serum (FBS, Thermo Fisher Scientific), 100 U/ml penicillin and 100 µg/ml streptomycin (Thermo Fisher Scientific) were added to all media. HEK-293T cells were cultured in Dulbecco’s Modified Eagle medium (DMEM, Thermo Fisher Scientific) and BT-474 cells were grown in Roswell Park Memorial Institute (RPMI, Thermo Fisher Scientific) 1640 medium with 4.5 g/l Glc.

### Transfection

For transient transfection of HEK-293T cells, Lipofectamine 2000 (Thermo Fisher Scientific) was used. HEK-293T cells (1.6 × 10^6^ cells/flask) were seeded in T-25 cell culture flasks and transfected with a total amount of 4 µg of plasmid on the following day. BT-474 cells were transfected with siRNA (96-well plate: 7.5 pmol; 12-well plate: 75 pmol) directed against HCA_2_ mRNA (siHCA_2_-I; siHCA_2_-II; Table S1), fluorescently labeled siRNA (siTC) or scrambled negative control siRNA (siNC) (OriGene) using SAINT-sRNA (Synvolux). A total of 11.5 µl (96-well plate) or 115 µl (12-well plate) of the siRNA/SAINT-sRNA mix was added to the medium. BT-474 cell RNA was prepared for determination of siRNA knockdown efficiency 48 h after siRNA transfection.

### RNA preparation, reverse transcription, real-time quantitative PCR

BT-474 cell RNA was isolated using the ReliaPrep RNA Cell Miniprep System (Promega) following the company’s protocol, and the yielded RNA was stored at − 80 °C. The RNA concentration was determined with a NanoDrop ND-1000, and an additional DNase I (NEB) digestion was performed at 37 °C for 30 min before reverse transcription to remove any remaining DNA. Afterward, DNase I was inactivated by the addition of EDTA (5 mM) and incubation at 75 °C for 10 min. Finally, the iScript cDNA Synthesis Kit (Bio-Rad) was used to transcribe RNA (500 ng) into cDNA. The cDNA solution was diluted with nuclease-free water to a final volume of 70 µl. Real-time quantitative PCR (RT-qPCR) was performed with 1 µl cDNA, 1 µl primer mix with sense and antisense primers (400 nM of each primer), 5 µl Luna Universal qPCR Master Mix (NEB) and 5 µl nuclease-free water on the CFX Connect Real-Time PCR Detection System (Bio-Rad). RT-qPCR was initiated with the activation of the DNA polymerase at 95 °C for 2 min followed by 40 cycles of denaturation at 95 °C for 15 s and primer annealing and elongation at 60 °C for 30 s. Fluorescence was measured at the end of each annealing/elongation step. Melting curves were recorded from 55 to 95 °C (0.5 °C increment, 5 s per step) and showed one single peak for each primer pair. Primers were designed with Primer-Blast (http://www.ncbi.nlm.nih.gov/tools/primer-blast/) and purchased from Microsynth Seqlab. For calculation of Δc_t_ values, the mRNA expression of the reference genes ACTB and RPS18 was determined.

### Cloning of HCA_2_

The cloning of human HCA_2_ was previously described [17]. The identity and correctness of the construct were confirmed by sequencing (SeqLab).

### Determination of HCA_2_ protein knockdown efficiency

HEK-293T cells were cotransfected with siHCA_2_ or siNC and plasmids encoding Nterminally HA- and C-terminally FLAG-tagged HCA_2_ as described in [18]. HCA_2_ cell surface expression was determined using an indirect cellular ELISA as described in [19].

### Seahorse metabolic analyses

Metabolic analyses were performed using the Seahorse XF Cell Mito Stress Test Kit and the XFe96 Analyzer (Seahorse Bioscience). BT-474 cells were seeded in Poly-l-Lysine (Sigma Aldrich) coated XF96 cell culture microplates (1.5 × 10^4^ cells/well) and incubated at 37 °C and 5% CO_2_. To analyze the role of HCA_2_ in cancer cell metabolism, cells were transfected with siHCA_2_ (siHCA_2_-I; siHCA_2_-II) or siNC 24 h before the assay. Niacin-free RPMI medium (Cell Culture Technologies) supplemented with (A) 10 mM Glc, 0.5 M L-carnitine and 10 mM BSA, (B) 10 mM Glc, 0.5 M L-carnitine and 10 mM BSA-palmitate or (C) 10 mM Glc and 2 mM Gln was used as assay medium.

On the day of the assay, the cells were washed and incubated with the assay medium in a CO_2_-free incubator at 37 °C. Subsequently, BT-474 cells were incubated with vehicle or 200 µM MMF (Sigma Aldrich) in the respective assay medium. The respiratory chain inhibitors oligomycin A (OA), carbonyl cyanide-4 (trifluoromethoxy) phenylhydrazone (FCCP) and rotenone/antimycin A (Rot/AA) were used for the determination of different mitochondrial parameters and loaded into the ports of the sensor cartridge following the manufacturer’s protocol (Seahorse Bioscience). The final well concentrations in the experiments were: 1.5 µM OA, 2 µM FCCP and 0.5 µM Rot/AA. The electron transport chain (ETC) consists of complex I (inhibited by rotenone), complex II, complex III (inhibited by antimycin A) and complex IV. The electrochemical proton gradient generated by the ETC is used for ATP generation by complex V/ATP synthase (inhibited by oligomycin A). FCCP is a chemical uncoupler that disrupts the proton gradient and mitochondrial membrane potential allowing maximal oxygen consumption. After 15 to 30 min calibration of the cartridge in the XFe96 Analyzer, the utility plate was replaced with the cell culture microplate and the assay was started. The oxygen consumption rate (OCR) and extracellular acidification rate (ECAR) were determined every 6 min for 18 min under basal conditions as well as after the consecutive injection of OA, FCCP and Rot/AA. After the assay, the cells were stained with Hoechst 33342 (1 µg/ml, Sigma Aldrich), and the cell number per well was determined with a Celigo Image Cytometer (Nexcelom/Cenibra). Finally, OCR and ECAR were normalized to 10^4^ cells.

### Liquid chromatography-mass spectrometry (LC-MS) measurement

One day prior to transfection, BT-474 cells were seeded in 6-well plates (4.5 × 10^5^ cells/well) in RPMI 1640 containing 10% FBS, 4.5 g/l Glc, 2 mM Gln, 5 mM HEPES, and 1 mM sodium pyruvate. The medium was changed prior to and 24 h after transfection, and the cells were transfected as described above. 48 h after transfection, the plates were placed on ice, the medium was removed from the plates, and the cells were washed with 1 ml of ice-cold PBS.

For stimulation experiments, BT474 cells were seeded in 12-well plates (2 × 10^5^ cells/well) and cultured for 72 h. On the day of stimulation, the medium was removed, and 1 ml of 100 µM MMF in serum-free RPMI 1640 or medium alone was added to the cells and incubated for 30 min at 37 °C. Thereafter, the plates were placed on ice, and the agonist solution was removed.

For both siRNA and stimulation experiments, 0.5 ml of ice-cold MeOH:H_2_O (90:10) and 1 scoop of glass beads were subsequently directly added to each well. Cells were scraped using a plastic cell scraper. Subsequently, the solution and the beads were transferred to prechilled tubes, extracted for 2 min at 30 Hz and centrifuged for 10 min at 14.000 rpm at 4 °C. Then, 400 µl of supernatant was transferred to LC-MS glass vials, dried in a speed vacuum concentrator and stored at − 20 °C until analysis. Samples were dissolved in 20 µl 50:50 MeOH:H_2_O, of which 2 µl was injected into the Agilent 1290 LC-system connected to a 6550 Agilent Q-TOF mass spectrometer, and an electrospray ionization (ESI) source was used. Data were collected in positive and negative ionization modes. The ESI (Agilent Jetstream) settings were as follows: gas temperature 300 °C, gas flow 8 l/min, nebulizer pressure 40 psi, sheet gas temperature 350 °C, sheet gas flow 11, Vcap 4000, fragmentor 100, Skimmer1 45 and Octapole RFPeak 750. Medium metabolites were separated using reversed-phase chromatography (Kinetex C18, 100 mm * 2.1 mm, 2.6 µM 100 Å, Phenomenex). For reversed-phase elution, solvents were prepared as follows: (A) H_2_O, 0.1% formic acid, (B) 75:25 acetonitrile: isopropanol, 0.1% formic acid. All solvents were of HPLC grade. Linear gradients were devised as follows for reversed-phase separation (0.5 ml/minute): minute 0: 5% B, minute 8: 95% B, minute 10: 95% B, minute 10.2: 5% B, minute 12: 5% B. Data were analyzed using Mass Profiler Professional (MPP) (Agilent) using the “find by formula” function with a match tolerance for masses of 10 ppm and for retention times of 0.35 min. The list of all compounds analyzed, including sum formulas, monoisotopic masses and fold changes, can be found in Table S2. Metabolites were identified using synthetic standards obtained from Sigma Aldrich and Santa Cruz Biotechnology by comparing accurate mass, retention time and, in some cases, MS/MS spectra.

### Data analyses

All data were statistically analyzed and visualized using GraphPad Prism 7 for Windows (GraphPad Software, San Diego California USA, www.graphpad.com). Detailed information about statistical analyses is included in each figure legend.

## Results

### In the presence of glucose, knockdown of HCA_2_ caused a decrease in mitochondrial respiration and ECAR

**Fig. 1:**
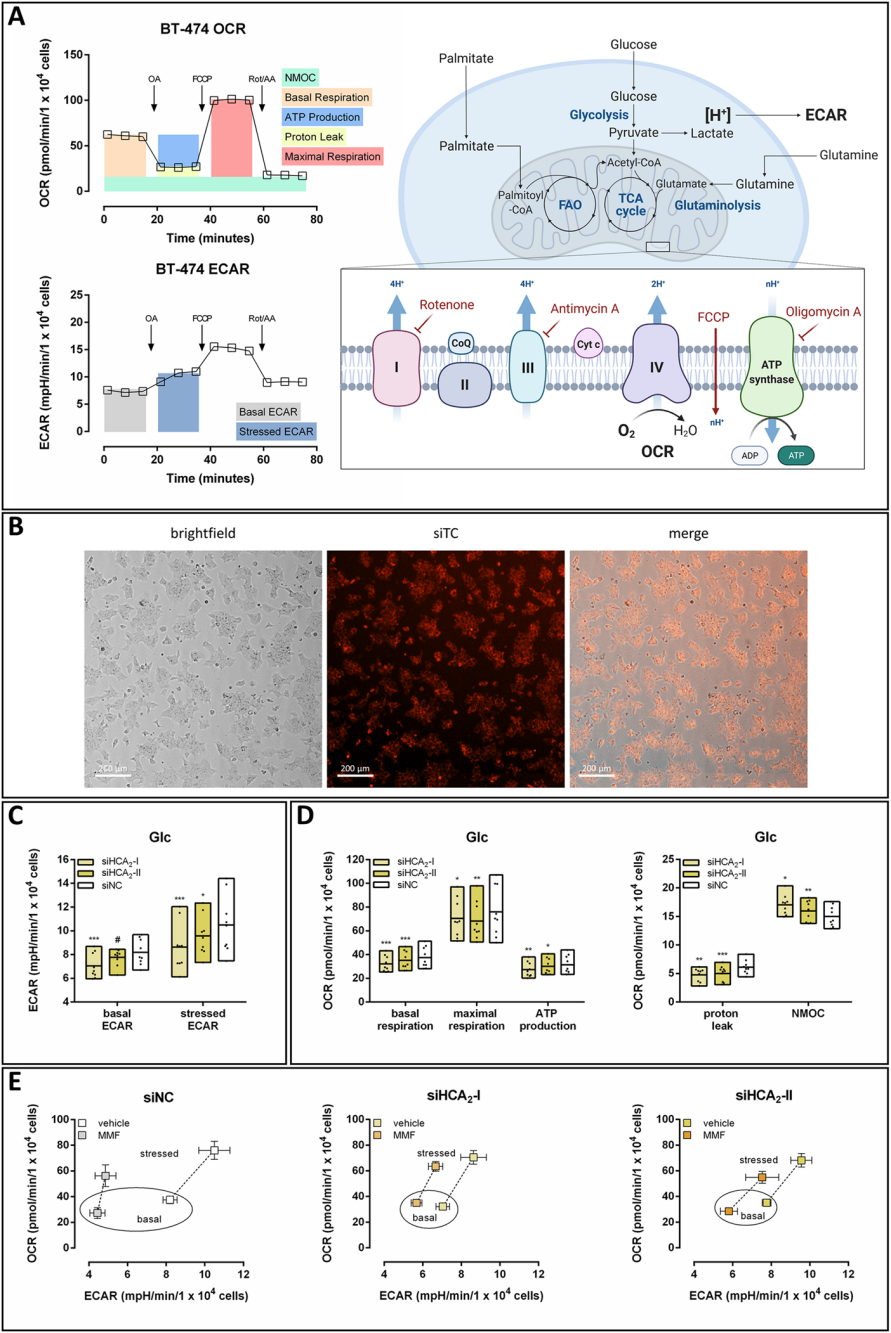
Knockdown of HCA_2_ was accompanied by a decrease in mitochondrial respiration and ECAR in BT-474 cells with glucose as the only energy substrate. **A** The oxygen consumption rate (OCR) and extracellular acidification rate (ECAR) of BT-474 cells were measured using a Seahorse XFe96 Analyzer. With the help of specific inhibitors of the electron transport chain, different parameters of mitochondrial respiration and non-mitochondrial oxygen consumption (NMOC) were determined. **B** BT-474 cells were transfected with fluorescently labeled siRNA (siTC), and images were taken 24 h after transfection to determine siRNA transfection efficiency. **C–E** 24 h after BT474 cells were transfected with siNC or two different HCA_2_-specific siRNAs (siHCA_2_-I; siHCA_2_-II), OCR and ECAR were measured in the presence of glucose (Glc). Knockdown of HCA_2_ caused a decrease in **C** ECAR as well as **D** basal and maximal respiration, oxygen consumption coupled to ATP production and proton leak. **E** The metabolic phenotype and potential (dotted line) of BT-474 cells were determined in the absence and presence of the HCA_2_ agonist monomethyl fumarate (MMF). Stimulation with MMF caused an HCA_2_-dependent shift to a less energetic phenotype. Data are shown as mean ± SEM (**A**, **E**) or floating bars (min to max; **C**, **D**) of three time points of three independent experiments, each carried out in six technical replicates. Statistical analyses were performed using repeated measures one-way ANOVA. ^#^P ≤ 0.1; *P ≤ 0.05; **P ≤ 0.01; ***P ≤ 0.001

We performed metabolic analyses with the Seahorse XFe96 Analyzer, which allows for simultaneous detection of the oxygen consumption rate (OCR) and the extracellular acidification rate (ECAR). The consecutive injection of the respiratory chain inhibitors oligomycin A (OA), rotenone and antimycin A (Rot/AA) and the uncoupler FCCP enabled the determination of glycolytic flux, different parameters of mitochondrial respiration and non-mitochondrial oxygen consumption (NMOC; Fig. 1A). BT-474 cells were transfected with scrambled negative control siRNA (siNC) or two different HCA_2_-specific siRNAs (siHCA_2_-I; siHCA_2_-II), and metabolic analyses were performed 24 h after transfection. A fluorescently labeled siRNA (siTC) was used to monitor transfection efficiency revealing successful transfection of BT-474 cells (Fig. 1B). Knockdown of HCA_2_ at the mRNA level was confirmed using RT-qPCR with HCA_2_ mRNA expression of 47% (siHCA_2_-I) and 39% (siHCA_2_II) compared to siNC (Figure S1A). Furthermore, we verified the knockdown at the protein level by cotransfection of HEK-293T cells, which do not endogenously express HCA_2_, with siHCA_2_ and plasmids encoding HA-tagged HCA_2_.

We confirmed a significant reduction (~ 43%) in cell surface HCA_2_ protein levels in a surface ELISA with the help of a peroxidase-coupled anti-HA antibody (Figure S1B).

In the medium containing only Glc as the main energy substrate, we found that knockdown of HCA_2_ caused a reduction in ECAR, which primarily reflects the production of lactate and, thus, the glycolytic rate (Fig. 1C). Furthermore, metabolic analyses revealed decreased basal and maximal respiration, oxygen consumption coupled to ATP production and proton leak in siHCA_2_ transfected BT-474 cells, accompanied by an increase in NMOC (Fig. 1D).

In addition, we determined the metabolic phenotype under basal and stressed conditions in the absence and presence of the HCA_2_-specific agonist monomethyl fumarate (MMF; Fig. 1E) [10, 20]. Stressed ECAR was measured after the injection of OA, which inhibits mitochondrial ATP synthesis (Fig. 1A). Thus, at this point, cells rely merely on non-mitochondrial processes such as glycolysis for the generation of ATP. Stressed OCR (maximal respiration) is determined after injection of FCCP, which is an uncoupling agent that disrupts the mitochondrial membrane potential, leading to an uninhibited flow of electrons and a maximal consumption of oxygen (Fig. 1A). These analyses revealed that stimulation of BT-474 cells with MMF in the presence of HCA_2_ (siNC) causes a shift towards lower OCR and ECAR under both basal and stressed conditions, reflecting a less energetic phenotype (Fig. 1E). This effect was highly diminished in siHCA_2_ transfected BT-474 cells, highlighting the specificity of the HCA_2_ agonist MMF (Fig. 1E). Accordingly, the metabolic potential, i.e. the cells’ ability to meet an energy demand via mitochondrial respiration and glycolysis (distance and slope of the dotted line connecting basal and stressed data points) was decreased in an HCA_2_-dependent manner upon stimulation with MMF (Fig. 1E).

### In the presence of glucose and glutamine, knockdown of HCA_2_ caused a decrease in stressed ECAR only

**Fig. 2:**
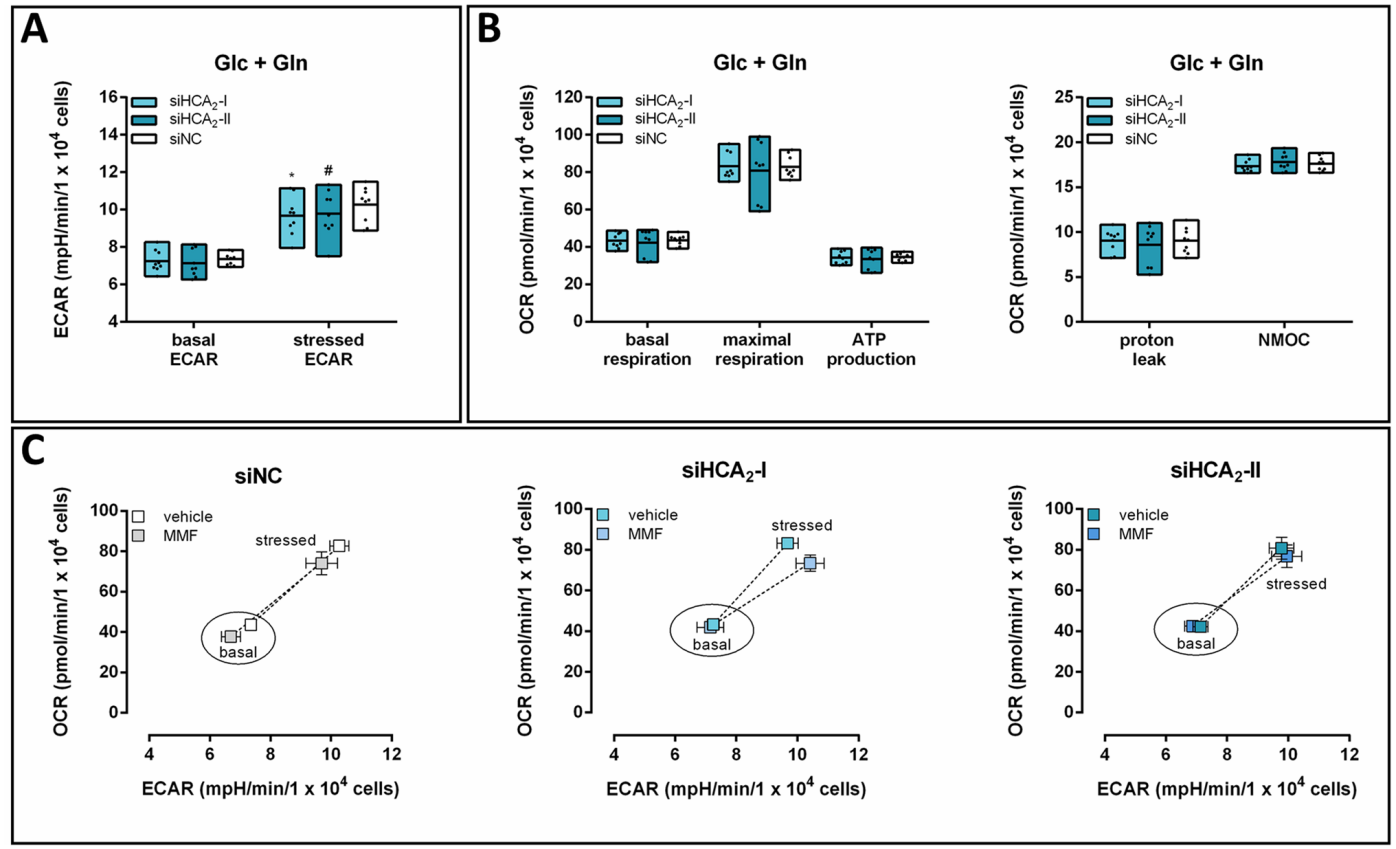
Reduction in stressed ECAR in siHCA_2_ transfected cells in the presence of glucose and glutamine. 24 h after BT-474 cells were transfected with siNC or two different siRNAs targeting HCA_2_ (siHCA_2_-I; siHCA_2_-II), OCR and ECAR were measured in the presence of glucose and glutamine (Glc + Gln). Knockdown of HCA_2_ caused a decrease in **A** stressed ECAR, while **B** mitochondrial respiration and non-mitochondrial oxygen consumption (NMOC) remained unaffected. **C** The metabolic phenotype and potential (dotted line) of BT-474 cells were determined in the absence and presence of the HCA_2_ agonist monomethyl fumarate (MMF). Stimulation with MMF caused an HCA_2_-dependent shift to a less energetic phenotype. Data are shown as floating bars (min to max; **A**, **B**) or mean ± SEM (**C**) of three time points of three independent experiments, each carried out in six technical replicates. Statistical analyses were performed using repeated measures one-way ANOVA. ^#^P ≤ 0.1; *P ≤ 0.05

In the medium containing both Glc and Gln, siHCA_2_ transfected cells exhibited only under stressed conditions a lower ECAR (Fig. 2A), while the respirational parameters remained unaffected (Fig. 2B). The presence of MMF shifted both the basal and stressed phenotypes towards lower OCR and ECAR, which was not observed in BT-474 cells with knockdown of HCA_2_ (Fig. 2C). This suggests a lower metabolic rate upon stimulation of the receptor under both basal and stressed conditions. However, the metabolic potential remained unaffected by stimulation with MMF independent of the presence of HCA_2_ (Fig. 2C).

### Mitochondrial respiration was decreased in cells with HCA_2_ knockdown when palmitate was available in addition to glucose

**Fig. 3:**
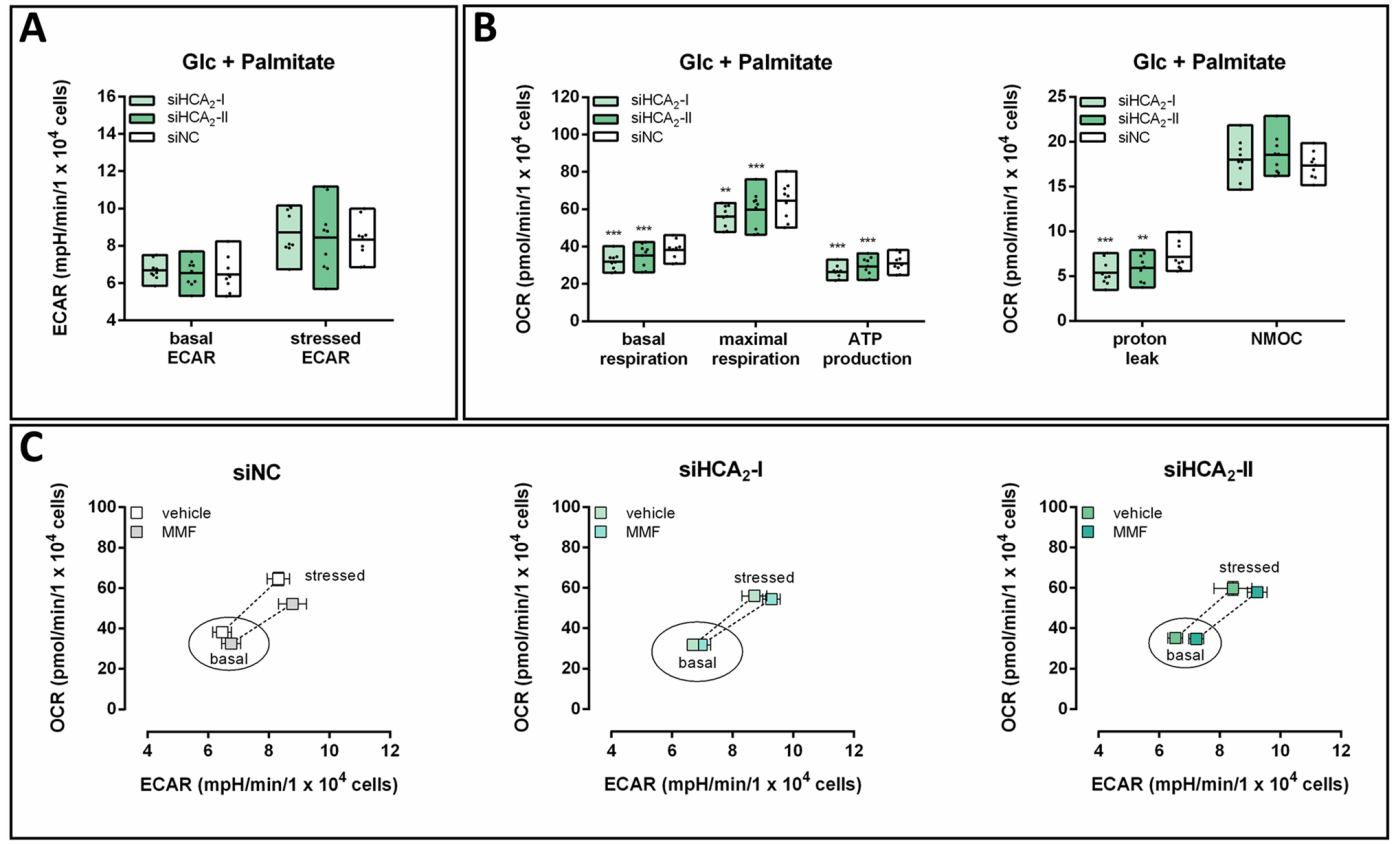
Mitochondrial respiration was reduced in cells with knockdown of HCA_2_ with palmitate and glucose as energy substrates. 24 h after BT-474 cells were transfected with siNC or two different siRNAs targeting HCA_2_ (siHCA_2_-I; siHCA_2_-II), OCR and ECAR were measured in the presence of glucose and palmitate (Glc + palmitate). Knockdown of HCA_2_ **A** did not affect ECAR, but **B** caused a decrease in mitochondrial respiration. **C** The metabolic phenotype and potential (dotted line) of BT-474 cells were determined in the absence and presence of the HCA_2_ agonist monomethyl fumarate (MMF). Stimulation with MMF caused an HCA_2_-dependent shift to a less aerobic phenotype under stressed conditions. Data are shown as floating bars (min to max; **A**, **B**) or mean ± SEM (**C**) of three time points of three independent experiments, each carried out in six technical replicates. Statistical analyses were performed using repeated measures one-way ANOVA. **P ≤ 0.01; *** P ≤ 0.001

At the beginning of the TCA cycle, acetyl-CoA reacts with oxaloacetate to generate citrate. Acetyl-CoA may originate from glucose, amino acid or fatty acid metabolism [2]. Palmitate serves as a substrate for FAO resulting in acetyl-CoA which then may be further metabolized in the TCA cycle. To analyze the effect of HCA_2_ knockdown or stimulation with MMF on FAO, we determined OCR and ECAR in the presence of Glc and palmitate. Both basal and stressed ECAR remained unaffected by knockdown of HCA_2_ in the presence of palmitate (Fig. 3A). However, we found that in siHCA_2_ transfected BT-474 cells basal and maximal respiration, as well as ATP-linked oxygen consumption and proton leak, were significantly reduced (Fig. 3B). Again, we tested whether stimulation of BT-474 cells with 200 µM MMF affects the metabolic phenotype in an HCA_2_-dependent manner. These analyses revealed that in Glc- and palmitate-containing medium, the metabolic phenotype in MMF versus unstimulated siNC transfected cells differed only under stressed conditions (Fig. 3C). This effect was attenuated in siHCA_2_ transfected cells. The metabolic potential was not affected by the presence of MMF under these conditions (Fig. 3C).

### Knockdown of HCA_2_ caused changes in the mRNA expression of genes encoding key metabolic enzymes and the metabolite profile of BT-474 cells

**Fig. 4:**
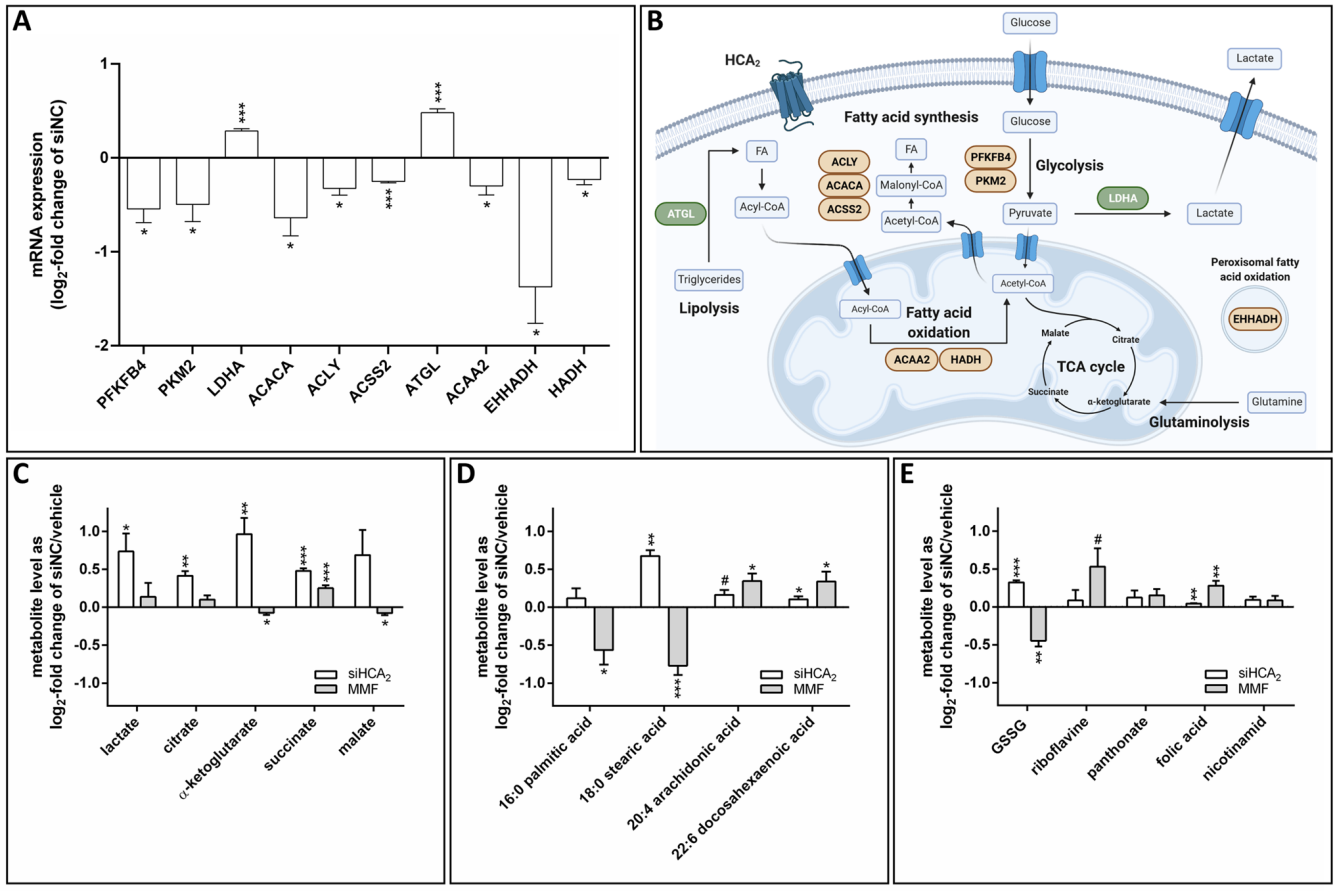
Knockdown of HCA_2_ induced changes in the expression of metabolic enzymes and the metabolite profile of BT-474 cells. 48 h after BT-474 cells were transfected with siNC or siRNAs targeting HCA_2_ (siHCA_2_), the mRNA expression of metabolic enzymes (**A**, **B**) was determined by RT-qPCR experiments, and the metabolite levels (**C–E**) were measured using LC-MS. Additionally, untransfected BT-474 cells were stimulated with 200 µM monomethyl fumarate (MMF) before LC-MS analysis. **A** Knockdown of HCA_2_ caused changes in the mRNA expression of metabolic enzymes involved in glycolysis, lipolysis, fatty acid oxidation and synthesis. **B** Main metabolic pathways used for energy generation and macromolecule synthesis with changes in the mRNA expression of key metabolic enzymes caused by knockdown of HCA_2_ (green: increased expression; red: decreased expression). **C–E** siRNA mediated knockdown (siHCA_2_) or stimulation of HCA_2_ with MMF led to changes in the levels of glycolysis and TCA cycle metabolites (**C**), fatty acids (**D**), oxidized glutathione (GSSG) and vitamins (**E**). Data are shown as mean ± SEM of n = 3 independent experiments. Statistical analyses were performed using an unpaired t-test. ^#^P ≤ 0.1; *P ≤ 0.05; **P ≤ 0.01; ***P ≤ 0.001

To test how the results of the metabolic phenotype analyses are reflected in mRNA expression changes of genes encoding enzymes that are involved in central metabolic processes, we performed RT-qPCR measurements. The expression of 37 genes involved in central metabolic pathways was analyzed in BT-474 cells that were transfected with siNC or siHCA_2_. 10 of these genes displayed a reproducible differential expression upon knockdown of HCA_2_ (Fig. 4A, B).

Knockdown of HCA_2_ caused a reduction in the mRNA expression levels of the enzymes 6-phosphofructo-2-kinase/fructose-2,6-bisphosphatase 4 (PFKFB4) and pyruvate kinase M2 (PKM2), which are important for the regulation of glycolysis (Fig. 4A) [21, 22]. Furthermore, the mRNA expression of lactate dehydrogenase A (LDHA) was elevated in siHCA_2_ transfected cells (Fig. 4A). The mRNA expression of genes encoding cytosolic proteins involved in FAS, i.e. acetyl-CoA carboxylase 1 (ACACA), ATP citrate lyase (ACLY) and acetyl-CoA synthetase 2 (ACSS2) were reduced upon knockdown of HCA_2_ (Fig. 4A). Similarly, the mRNA expression of genes involved in mitochondrial FAO, i.e. acetyl-CoA acyltransferase 2 (ACAA2), hydroxyacyl-CoA dehydrogenase (HADH), and peroxisomal oxidation of FAs (enoyl-CoA, hydratase/3-hydroxyacyl-CoA dehydrogenase (EHHADH)) were significantly lower in siHCA_2_ transfected compared to siNC transfected BT-474 cells (Fig. 4A). However, knockdown of HCA_2_ caused an increase in the mRNA expression of adipose triglyceride lipase (ATGL), an enzyme that hydrolyzes triacylglycerols to diacylglycerols, the first reaction of lipolysis (Fig. 4A) [23].

One of the aims of this study was to investigate whether and how the metabolic phenotype is reflected in transcriptional changes and metabolite levels determined by global metabolomics applying a general LC-MS methodology. Using a list of 170 standards run on the same LC-MS system as the cell samples, we extracted peak height data for these metabolites if detectable in the samples. Before the LC-MS measurements, BT-474 cells were transfected with siHCA_2_ or siNC. Moreover, untransfected cells were treated with vehicle or the HCA_2_ agonist MMF. In each experiment, siRNA transfection and stimulation with MMF were carried out three times, each in five replicates. Only 34 compounds were reliably detected in all experiments (Table S2). Of these 34 compounds, three (α-ketoglutarate, stearic acid, GSSG) showed contrary changes when comparing siHCA_2_ as x-fold of siNC versus MMF as x-fold of vehicle (Fig. 4C–E). Altogether, 14 metabolites exhibited significant differences in siHCA2 versus siNC transfected cells and 19 in unstimulated versus MMF stimulated BT-474 cells, including the previously mentioned three metabolites (Fig. 4C–E, Figure S1C, S1D).

LC-MS analysis showed a significant increase in the glycolytic and TCA cycle metabolites lactate, citrate, α-ketoglutarate and succinate upon knockdown of HCA_2_ compared to siNC transfected cells (Fig. 4C). Stimulation with MMF caused reduced relative levels of α-ketoglutarate and malate but elevated levels of succinate when compared to unstimulated cells (Fig. 4C). Furthermore, in cells with HCA_2_ knockdown, the relative levels of stearic acid, arachidonic acid and docosahexaenoic acid were increased in comparison to siNC transfected cells (Fig. 4D). The opposite was true for the comparison of MMF stimulated versus unstimulated cells for palmitic acid and stearic acid (Fig. 4D). Similarly, the relative level of oxidized glutathione (GSSG), an indicator of oxidative stress, showed a reduction upon stimulation with MMF compared to unstimulated cells, while the opposite was true for cells with knockdown of HCA_2_ versus siNC transfected cells (Fig. 4E).

## Discussion

Our present study aimed to determine the value of combining live-cell metabolic phenotype analyses with transcriptional analyses and global metabolomics. Metabolomics is increasingly used as a systematic measurement of diverse metabolites, such as nutrients and signaling mediators, in blood or tissue samples to identify potential disease/cancer biomarkers or even drivers of tumorigenesis [24–26]. Similarly, transcriptomics is applied to identify molecular cancer subtypes for more effective development of anti-cancer therapeutics [27]. Here, we evaluate to what extent the metabolic phenotype is reflected in changes in the metabolite and mRNA levels of genes involved in central metabolic pathways. We used the stimulation and knockdown of HCA_2_ as one selected Gα_i_-coupled receptor in the well-characterized BT-474 cell line as a very simplified model. HCA_2_ itself is not directly involved in cellular metabolism. With this intentionally chosen simplified in vitro approach, we eliminated as many variable parameters as possible to ensure that our analyses indeed reveal HCA_2_-regulated metabolic pathways. HCA_2_ has been suggested to act as a tumor suppressor [11–13] and we hypothesized that our approach will enable the identification of a set of metabolic and transcriptional markers associated with HCA_2_ function. However, future analyses have to test in an in vivo setting or in patient-derived pathology slices the adaptability of our results in a more complex setting that also takes parameters such as the tumor microenvironment into account.

In our setup, we performed experiments in non-substrate-limited medium for both HCA_2_ knockdown and MMF stimulation. Transcriptional analyses were performed using RT-qPCR for selected genes encoding enzymes involved in central metabolic processes, which have previously been shown to be relevant in a cancer cell context. In contrast to most transcriptomics and metabolomics, we repeated the experiment with three different passages of the BT-474 cell line with the indicated replicates to obtain a better understanding of the reproducibility of these analyses. Thus, one would expect a high reproducibility under controlled laboratory conditions with a cell line as a biological sample that is either transfected with siRNA or stimulated with the HCA_2_ agonist. We found that only 34 metabolites (Table S2) were reliably detected in all LC-MS experiments carried out. This was rather surprising since each experiment itself would have yielded up to 100 metabolites. In our view, this variability within the in vitro setting must be considered when interpreting metabolomics of plasma or other body fluids to identify cancer biomarkers. Here, we found that metabolite levels may be very variable even under strictly controlled laboratory conditions using a cell line as a model system. Further, to ensure that the observed changes were indeed due to effects mediated by HCA_2_, we were particularly interested in how many metabolites exhibit a contrarily regulated pattern when comparing cells with knockdown of HCA_2_ to those stimulated with MMF. This is important since MMF may have effects independent of HCA_2_. It is known that MMF elicits several cellular effects, such as the modulation of cellular glutathione content, which impacts responses to oxidative stress, or the modulation of oxidative stress-sensitive transcription factors, such as hypoxia-inducible factor-1α (reviewed in [10]). Our live-cell metabolic analyses revealed that the presence of different energy substrates differentially affects the determined metabolic phenotype regulated by HCA_2_. However, a link between HCA_2_ and an increased glycolytic flux was observed if no exogenous palmitate was available as an energy substrate (Fig. 1C). This is in line with our finding that knockdown of HCA_2_ resulted in reduced mRNA expression of the glycolytic enzymes PFKFB4 and PKM2 (Fig. 4A, B). Both PFKFB4 and PKM2 are upregulated in different types of cancer and have been shown to promote the Warburg effect as well as glycolytic flux in cancer cells [21, 22]. This elevated expression of PFKFB4 or PKM2 is associated with poor prognosis in different types of tumors suggesting that both enzymes are prognostic markers [21, 28]. Furthermore, LDHA mRNA levels were increased in cells with HCA_2_ knockdown, and LC-MS analyses revealed that intracellular lactate levels were also increased (Fig. 4A, C). LDHA converts pyruvate to lactate at the end of glycolysis, thereby reducing further aerobic metabolization in the mitochondria and contributing to aerobic glycolysis [29]. These results indicate that reduced HCA_2_ expression is associated with decreased glycolytic flux but at the same time an elevated conversion of pyruvate to lactate. Therapeutically, this may be exploited by using a combination therapy of HCA_2_ agonists with inhibitors of glycolysis, such as 2-deoxyglucose (reviewed in [30]).

With Glc as the main available energy substrate, mitochondrial respiration and ATP production were higher when HCA_2_ was expressed, while NMOC was decreased (Fig. 1D). With the additional availability of Gln, these effects of HCA_2_ on respirational parameters were abolished (Fig. 2B). Gln is a non-essential amino acid that is used in energy metabolism to fuel the TCA cycle anaplerotically after its conversion to α-ketoglutarate during glutaminolysis [31]. Thus, the utilization of Gln as fuel for mitochondrial metabolism might compensate for the observed effects on mitochondrial respiration in the presence of Glc alone (Fig. 1D). This is further supported by increased levels of the TCA cycle intermediates α-ketoglutarate and succinate that were detected in BT-474 cells with reduced HCA_2_ expression (Fig. 4C).

With the FA palmitate available in addition to Glc, HCA_2_ still caused increased mitochondrial respiration and ATP production (Fig. 3B). With both Glc and palmitate as energy substrates, the TCA cycle is fueled either by pyruvate from glycolysis or acetyl-CoA from FAO. Since no HCA_2_-dependent difference in ECAR was detected in the presence of palmitate (Fig. 3A), the HCA_2_ mediated increase in mitochondrial respiration is likely caused by the regulation of palmitate oxidation. Our analyses of transcriptional changes encoding enzymes involved in lipolysis, FAO or FAS revealed that only ATGL, which catalyzes the first step of triglyceride hydrolysis, exhibited increased mRNA expression in siHCA_2_ transfected cells (Fig. 4A) [23]. Thus, HCA_2_ suppresses ATGL expression and thereby lipolysis in BT-474 cells, which is in line with the physiological role of HCA_2_ in adipocytes, where it inhibits lipolysis [5]. On the other hand, the mRNA expression of all tested enzymes involved in FAO or FAS was reduced upon knockdown of HCA_2_ (Fig. 4A). This suggests that the presence of HCA_2_ stimulates both FAS and FAO. The enzyme that links carbohydrate metabolism with FAS by converting citrate to acetyl-CoA is ACLY [32], while ACSS2 encodes the cytosolic enzyme that catalyzes the rate-limiting step of lipid synthesis [33]. Both ACLY and ACSS2 are transcriptionally induced by HCA_2_, and metabolomics revealed that relative citrate levels are lower in cells with HCA_2_ (siNC; Fig. 4C). One potential explanation may be that if HCA_2_ indeed stimulates FAS, more citrate is consumed. However, since we neither analyzed metabolic flux nor actual citrate turnover, this conclusion is rather speculative. An increased FAO in BT-474 cells with HCA_2_ expression (siNC) is in accordance with the higher mitochondrial respiration observed with Glc and palmitate as available energy substrates (Fig. 3B).

Metabolomics revealed that stimulation of HCA_2_ caused a decrease in the relative levels of the FAs palmitic and stearic acid, while the opposite was true for stearic acid in cells with knockdown of HCA_2_ (Fig. 4D). In combination with the results we obtained regarding HCA_2_-dependent lipid metabolism, this decrease may indicate that HCA_2_ causes a combination of an increased FAO rate while simultaneously inhibiting lipolysis.

Taken together, our study shows that HCA_2_ regulates glycolytic flux and FA metabolism in BT-474 cells, which is reflected in live-cell metabolic analyses but also changes in the transcriptome and metabolite levels. Furthermore, we conclude that combining these methods facilitates a greater understanding of the complex network of oncogenic signaling pathways, which induce characteristic cancer phenotypes by the regulation of key metabolic processes. Regarding HCA_2_, we assume that a better knowledge of the tumor-suppressing properties of HCA_2_ is of great value to exploit the specific metabolic weak spots as valuable targets in combination with HCA_2_ agonists.

## Conclusions

The present study revealed HCA_2_ as a regulator of BT-474 breast cancer cell metabolism dependent on the available energy substrates. The presence of HCA_2_ was associated with increased glycolytic flux and elevated fatty acid oxidation. These findings were reflected in transcriptional changes and alterations of certain metabolites as determined by global metabolomics. Thus, treatment with HCA_2_ agonists already used to treat diseases such as psoriasis or hyperlipidemia may prove useful in combination cancer therapy.

## Supplementary Information

Supplementary file 1. (DOCX 586 KB)

## Data Availability

The datasets used and/or analyzed during the current study are available from the corresponding author on reasonable request.

## Abbreviations

AA: Antimycin A
ACAA2: Acetyl-CoA acyltransferase 2
ACACA: Acetyl-CoA carboxylase 1
ACLY: ATP citrate lyase
ACSS2: Acetyl-CoA synthetase 2
ACTB: Beta-actin
ADP: Adenosine diphosphate
ATGL: Adipose triglyceride lipase
ATP: Adenosine triphosphate
BSA: Bovine serum albumin
CoA: Coenzyme A
CoQ: Coenzyme Q Ubiquinone
Cyt c: Cytochrome c
DMEM: Dulbecco’s Modified Eagle Medium
ECAR: Extracellular acidification rate
EHHADH: Enoyl-CoA, hydratase/3-hydroxyacyl-CoA dehydrogenase
ELISA: Enzyme-linked immunosorbent assay
ESI: Electrospray ionization
ETC: Electron transport chain
FA: Fatty acid
FAO: Fatty acid oxidation
FAS: Fatty acid synthesis
FBS: Fetal bovine serum
FCCP: Carbonyl cyanide-4 (trifluoromethoxy) phenylhydrazone
Glc: Glucose
Gln: Glutamine
GPCR: G protein-coupled receptor
GSSG: Oxidized glutathione
HADH: Hydroxyacyl-CoA dehydrogenase
HCA_2_: Hydroxycarboxylic acid receptor 2
LC-MS: Liquid chromatography-mass spectrometry
LDHA: Lactate dehydrogenase A
MMF: Monomethyl fumarate
MS: Multiple sclerosis
NMOC: Non-mitochondrial oxygen consumption
OA: Oligomycin A
OCR: Oxygen consumption rate
PBS: Phosphate-buffered saline
PFKFB4: 6-phosphofructo-2-kinase/fructose-2,6-bisphosphatase 4
PKM2: Pyruvate kinase M2
ppm: Parts per million
Q-TOF: Quadrupole time of flight
Rot: Rotenone
RPMI: Roswell Park Memorial Institute
RPS18: 40S ribosomal protein 18
RT-qPCR: Real-time quantitative polymerase chain reaction
TCA: Tricarboxylic acid
