# Supplementary material for: Combining metabolic phenotype determination with metabolomics and transcriptional analyses to reveal pathways regulated by hydroxycarboxylic acid receptor 2
Source: Discov Oncol. 2022 Jun 13;13:47. doi: 10.1007/s12672-022-00503-3 (PMC9192902; doi:10.1007/s12672-022-00503-3)
Supplement: Supplementary file 1 — (DOCX 586 KB) [file 12672_2022_503_MOESM1_ESM.docx]

## *Supplementary Material*

**Combining metabolic phenotype determination with metabolomics and transcriptional analyses to reveal pathways regulated by hydroxycarboxylic acid receptor 2**

Philipp Rabe^1^, Mareike Gehmlich^1^_,_ Anna Peters^1^, Petra Krumbholz^1^, Anders Nordström^2^, Claudia Stäubert^1^*

^1^ Rudolf Schönheimer Institute of Biochemistry, Faculty of Medicine, Leipzig University, Leipzig, Germany

^2^ Swedish Metabolomics Centre, Department of Forest Genetics and Plant Physiology, Swedish University of Agricultural Sciences, Linnaeus väg 6, 901 87 Umeå, Sweden

* To whom correspondence and requests for materials should be addressed:

Claudia Stäubert, Rudolf Schönheimer Institute of Biochemistry, Faculty of Medicine, University of Leipzig, Johannisallee 30, 04103 Leipzig, Germany, Tel.: +49-341-9722-157, Fax: +49-341-9722-159, Email: claudia.staeubert@medizin.uni-leipzig.de


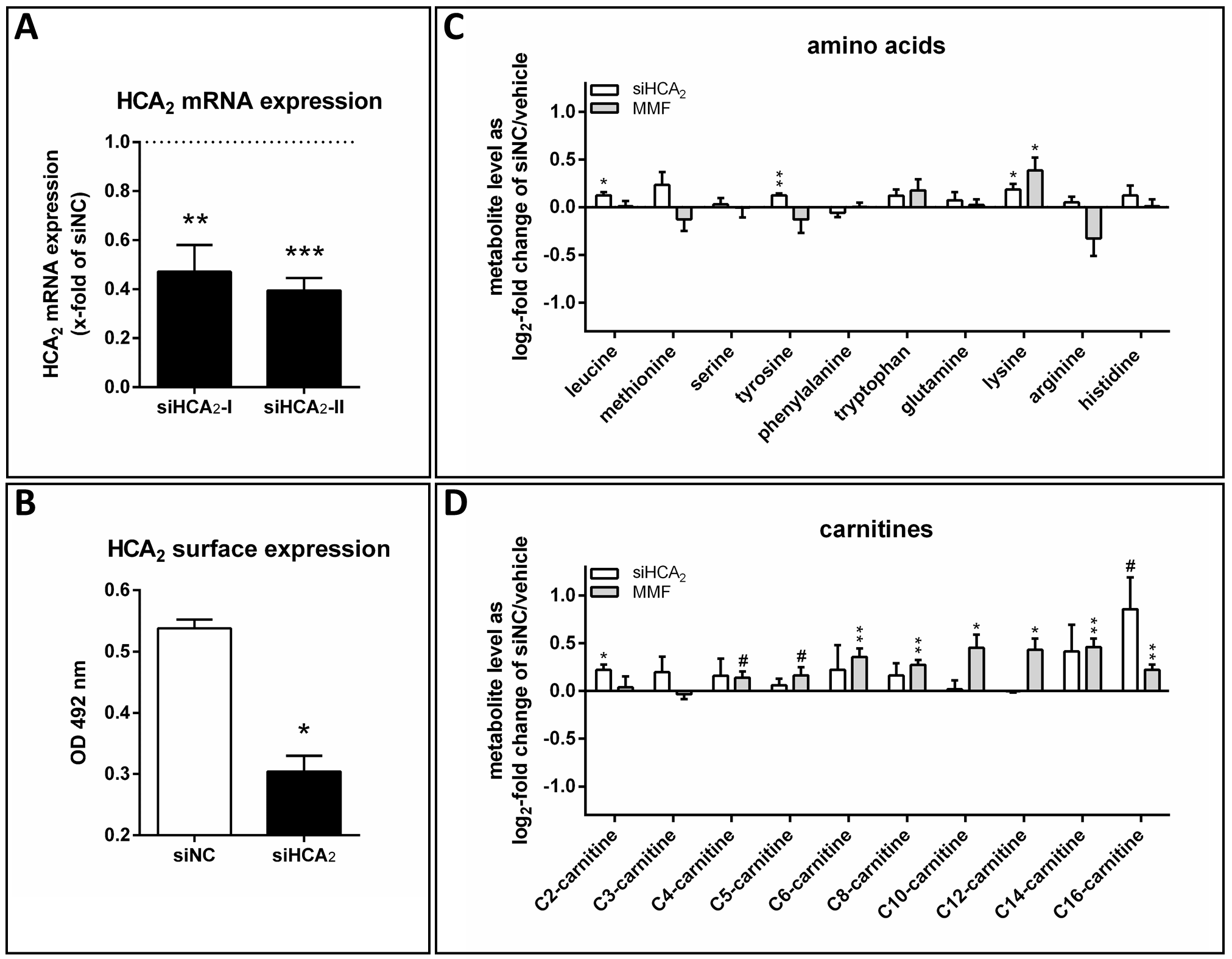


### **Figure S1: HCA_2_ mRNA and protein knockdown efficiency, amino acid and carnitine levels upon knockdown or stimulation of HCA_2_.**

A: BT-474 cells were transfected with siNC or siHCA_2_ and the reduction in HCA_2_ mRNA level was detected by qPCR. B: HEK293-T cells were cotransfected with a plasmid encoding an HA-tagged HCA_2_ and siHCA_2_ or siNC. After 48 h, a decrease in cell surface HCA_2_ protein expression was detected using an ELISA with a peroxidase-coupled anti-HA-antibody. OD was measured at 492 nm. C, D: siRNA mediated knockdown (siHCA_2_) or stimulation of HCA_2_ with 200 µM monomethylfumarate (MMF) caused changes in the levels of amino acids (C) and carnitines (D). Data are shown as mean ± SEM of n = 3 independent experiments. Statistical analyses were performed using an unpaired (A, C, D) or paired (B) t-test. # P ≤ 0.1; * P ≤ 0.05; ** P ≤ 0.01; *** P ≤ 0.001.

### **Table S1: List of all primers used for qPCR and siRNA sequences provided by Origene.** Sequences of the primer pairs (S: sense; AS: antisense) used in qPCR analyses and siRNA sequences provided by Origene.

| **Primer** | **Sequence (5’-3’)** |
| --- | --- |
| ACTB-1412-S | ACAATGTGGCCGAGGACTTT |
| ACTB-1519-AS | TGGGGTGGCTTTTAGGATGG |
| RPS18-106-S | GATGGGCGGCGGAAAATAG |
| RPS18-192-AS | GTCTGCTTTCCTCAACACCAC |
| HCAR2-266-S | TTTCCTGTTCAACCTGGCAGT |
| HCAR2-352-AS | CAGTCCCAACGCCTCACAT |
| PFKFB4-37-S | TGACACAGAACCCCCTGAAG |
| PFKFB4-140-AS | GAGAGTTGGGCAGTTGGTCA |
| PKM2-1246-S | GGAAGCCTGTCATCTGTGCT |
| PKM2-1390-AS | TCCCCTTTGGCTGTTTCTCC |
| LDHA-568-S | GTCAGCAAGAGGGAGAAAGCC |
| LDHA-723-AS | TTATCTTCCAAGCCACGTAGGTC |
| ACACA-8143-S | TTACCCCGACTCTTAGCCCA |
| ACACA-8298-AS | CTTGGCTCACAGTTTTGCCC |
| ACLY-1874-S | AACTTTGCCTCTCTCCGCTC |
| ACLY-2029-AS | TCCGATGATGGTCACTCCCT |
| ACSS2-1001-S | CAAGAGGCAGGGGATGAGTG |
| ACSS2-1122-AS | ATGTAGCCCCCAACTGTGTG |
| ATGL-707-S | AGATGTGCAAGCAGGGATACC |
| ATGL-864-AS | CGAGTAATCCTCCGCTTGGG |
| ACAA2-3-S | GTTTAGGGTGTTGGCGGAGA |
| ACAA2-145-AS | GAGCGGACTCTTTCCTCACA |
| EHHADH-139-S | CAACGCGATCAGTACGACTTT |
| EHHADH-245-AS | ATTTGCCCTCTGCTCCACAA |
| HADH-404-S | TCCTGGCAAAATCCAAAAAGGG |
| HADH-531-AS | CGTGCTGGTCGCTATGGT |
| **siRNA** |  |
| siHCA_2_-I | rArArCrCrUrCrUrCrCrUrUrArArArUrArArCrCrArUrGrCCA |
| siHCA_2_-II | rCrUrCrArCrArCrGrCrUrUrUrGrGrUrUrArArUrArUrCrUGT |

### **Table S2: Targeted list of compounds that were detected in siRNA transfected or MMF-stimulated BT-474 cells using LC-MS.** Determined peak height was normalized to siNC or unstimulated BT-474 cells, respectively (each set 1).

| monoisotopic mass | sum formula | name | siHCA_2_ | MMF |
| --- | --- | --- | --- | --- |
| **positive mode** |  |  |  |  |
| 204.1235 | C9H18NO4 | Acetylcarnitine | 1.17 ± 0.05 | 1.04 ± 0.08 |
| 218.1386 | C11H21NO4 | Propionylcarnitine | 1.03 ± 0.06 | 0.98 ± 0.03 |
| 231.1471 | C11H21NO4 | Butyrylcarnitine | 0.99 ± 0.04 | 1.11 ± 0.05 |
| 275.1369 | C12H21NO6 | Glutarylcarnitine | 1.01 ± 0.06 | 1.13 ± 0.06 |
| 259.1784 | C13H25NO4 | Hexanoylcarnitine | 0.98 ± 0.04 | 1.29 ± 0.08 |
| 287.2097 | C15H29NO4 | Octanoylcarnitine | 1.04 ± 0.08 | 1.21 ± 0.05 |
| 315.2410 | C17H33NO4 | Decanoylcarnitine | 1.01 ± 0.07 | 1.39 ± 0.13 |
| 343.2723 | C19H37NO4 | Dodecanoylcarnitine | 1.00 ± 0.01 | 1.36 ± 0.10 |
| 371.3036 | C21H41NO4 | Tetradecanoylcarnitine | 1.14 ± 0.06 | 1.38 ± 0.08 |
| 399.3349 | C23H45NO4 | Hexadecanoylcarnitine | 1.58 ± 0.17 | 1.17 ± 0.05 |
| **negative mode** |  |  |  |  |
| 90.0316 | C3H6O3 | Lactic acid | 1.71 ± 0.28 | 0.89 ± 0.07 |
| 192.0270 | C6H8O7 | Citric acid | 1.34 ± 0.06 | 1.08 ± 0.04 |
| 146.0215 | C5H6O5 | α-Ketoglutaric acid | 1.97 ± 0.29 | 095 ± 0.02 |
| 118.0266 | C4H6O4 | Succinic acid | 1.39 ± 0.03 | 1.19 ± 0.03 |
| 134.0216 | C4H6O5 | Malic acid | 1.69 ± 0.34 | 0.95 ± 0.02 |
| 105.0426 | C3H7NO3 | Serine | 1.02 ± 0.05 | 1.01 ± 0.07 |
| 131.0946 | C6H13NO2 | Leucine | 1.09 ± 0.03 | 1.01 ± 0.04 |
| 146.0691 | C5H10N2O3 | Glutamine | 1.06 ± 0.06 | 1.02 ± 0.04 |
| 146.1055 | C6H14N2O2 | Lysine | 1.14 ± 0.05 | 1.21 ± 0.07 |
| 149.0511 | C5H11NO2S | Methionine | 1.19 ± 0.11 | 0.93 ± 0.08 |
| 174.1117 | C6H14N4O2 | Arginine | 1.04 ± 0.04 | 0.82 ± 0.10 |
| 155.0695 | C6H9N3O2 | Histidine | 1.06 ± 0.02 | 1.01 ± 0.01 |
| 165.0790 | C9H11NO2 | Phenylalanine | 0.97 ± 0.02 | 1.00 ± 0.03 |
| 181.0740 | C9H11NO3 | Tyrosine | 1.09 ± 0.01 | 0.93 ± 0.09 |
| 204.0899 | C11H12N2O2 | Tryptophan | 1.01 ± 0.04 | 1.05 ± 0.04 |
| 256.2402 | C16H32O2 | Palmitic acid (16:0) | 1.17±0.11 | 0.69 ± 0.09 |
| 284.2715 | C18H36O2 | Stearic acid (18:0) | 1.60 ± 0.09 | 0.59 ± 0.05 |
| 304.2402 | C20H32O2 | Arachidonic acid (20:4(n-6)) | 1.06 ± 0.02 | 1.36 ± 0.06 |
| 328.2402 | C22H32O2 | Docosahexaenoate 22:6(n-3) | 1.08 ± 0.03 | 1.36 ± 0.11 |
| 122.0480 | C6H6N2O | Nicotinamide | 1.07 ± 0.03 | 1.06 ± 0.05 |
| 219.1107 | C9H17NO5 | Pantothenate | 1.10 ± 0.04 | 1.17 ± 0.06 |
| 376.1383 | C17H20N4O6 | Riboflavin (Vitamin B2) | 1.06 ± 0.06 | 1.35 ± 0.20 |
| 441.1397 | C19H19N7O6 | Folic acid | 1.02 ± 0.01 | 1.22 ± 0.05 |
| 612.1520 | C20H32N6O12S2 | Glutathione oxidized (GSSG) | 1.25 ± 0.04 | 0.75 ± 0.04 |
